# A comprehensive review of the impact of COVID-19 on human reproductive biology, assisted reproduction care and pregnancy: a Canadian perspective

**DOI:** 10.1186/s13048-020-00737-1

**Published:** 2020-11-27

**Authors:** Mitko Madjunkov, Michal Dviri, Clifford Librach

**Affiliations:** 1 CReATe Fertility Centre, 790 Bay Street, Suite 1100, Toronto, M5G1N8 Canada; 2 Department of Obstetrics and Gynecology, University of Toronto, Toronto, Canada; 3 Institute of Medical Sciences, University of Toronto, Toronto, Canada; 4 Department of Physiology, University of Toronto, Toronto, Canada

**Keywords:** SARS-CoV-2, COVID-19, ART-assisted reproductive technologies, Pregnancy, Reproduction

## Abstract

Currently, the world is in the seventh month of the COVID-19 pandemic. Globally, infections with novel SARS-CoV-2 virus are continuously rising with mounting numbers of deaths. International and local public health responses, almost in synchrony, imposed restrictions to minimize spread of the virus, overload of health system capacity, and deficit of personal protective equipment (PPE). Although in most cases the symptoms are mild or absent, SARS-CoV-2 infection can lead to serious acute respiratory disease and multisystem failure. The research community responded to this new disease with a high level of transparency and data sharing; with the aim to better understand the origin, pathophysiology, epidemiology and clinical manifestations. The ultimate goal of this research is to develop vaccines for prevention, mitigation strategies, as well as potential therapeutics.

The aim of this review is to summarize current knowledge regarding the novel SARS CoV-2, including its pathophysiology and epidemiology, as well as, what is known about the potential impact of COVID-19 on reproduction, fertility care, pregnancy and neonatal outcome. This summary also evaluates the effects of this pandemic on reproductive care and research, from Canadian perspective, and discusses future implications.

In summary, reported data on pregnant women is limited, suggesting that COVID-19 symptoms and severity of the disease during pregnancy are similar to those in non-pregnant women, with pregnancy outcomes closely related to severity of maternal disease. Evidence of SARS-CoV-2 effects on gametes is limited. Human reproduction societies have issued guidelines for practice during COVID-19 pandemic that include implementation of mitigation practices and infection control protocols in fertility care units. In Canada, imposed restrictions at the beginning of the pandemic were successful in containing spread of the infection, allowing for eventual resumption of assisted reproductive treatments under new guidelines for practice. Canada dedicated funds to support COVID-19 research including a surveillance study to monitor outcomes of COVID-19 during pregnancy and assisted reproduction. Continuous evaluation of new evidence must be in place to carefully adjust recommendations on patient management during assisted reproductive technologies (ART) and in pregnancy.

## Introduction - COVID-19 epidemiology worldwide and in Canada

On December 29th, 2019, in Wuhan, the capital city of Hubei province of China, four cases of pneumonia of unknown etiology were reported. On Jan 12th, 2020, next generation sequencing analysis of the full-length genome from respiratory tract samples of the pneumonia cases indicated a novel coronavirus (CoV), previously not linked with infection in humans. On February 11th, 2020 the International Committee of Taxonomy of Viruses, recognized the etiology of this infection and named it ‘Severe acute respiratory syndrome coronavirus-2’ (SARS-CoV-2) and at the same time the World Health Organization (WHO) named the disease COVID-19 (https://www.who.int/emergencies/diseases/novel-coronavirus-2019/technical-guidance/naming-the-coronavirus-disease-(covid-2019)-and-the-virus-that-causes-it).

Epidemiologically, the first cases were linked to the wet seafood market in Wuhan, where the sale of animals that allowed transmission of the virus from the host bat reservoir through an, as yet not identified, animal host, facilitated the adaptation and capacity of this virus to jump species boundaries and infect humans (https://www.who.int/emergencies/diseases/novel-coronavirus-2019/technical-guidance/naming-the-coronavirus-disease-(covid-2019)-and-the-virus-that-causes-it, [1]). COVID-19 spread very quickly throughout the world, and on March 11th, 2020, the WHO declared it a pandemic. By September 29th, 2020, more than 33 million people have been infected worldwide, and 1,007,508 have died (https://www.worldometers.info/coronavirus/country/canada/). In Canada, as of that date, 155,301 have been infected with SARS-CoV-2, 141,885 are ‘closed’ cases (132,607 (~ 93.5%) have recovered, and 9278 (6.5%) have died), and 13,416 are active cases (99% (13,306) with mild disease and 1% (110) with severe or critical disease) (https://www.worldometers.info/coronavirus/country/canada/). The most severely affected provinces in Canada are Quebec and Ontario with 72,651 and 50,531 positive cases to date respectively, accounting for over 80% of the cases in Canada. In Quebec and Ontario there have been 5826 and 2840 deaths respectively, accounting for 93.4% of the all deaths in Canada (https://www.canada.ca/en/public-health/services/diseases/2019-novel-coronavirus-infection.html). The majority of Canadian COVID-19 related deaths have been in patients over 70 years of age (8242/29,050) (mortality rate of ~ 28%), with ~ 90% of all deceased patients being older than 70 years: 80+ years accounting for 71.3% of COVID-19 related deaths (6567/9210) and 70–80 years old for 18.2% (1675/9210). The distribution of deaths in patients in younger age groups are: ~ 7.3% (668/9210) for 60-69y age group, 3% (274/9210) for 40-59y, 0.26% (24/9210) for 20–39 y and 0.02% (2/9210) for the youngest group of 0–19 years (https://health-infobase.canada.ca/covid-19/epidemiological-summary-covid-19-cases.html#fn3). The high mortality rate in older people has been due to the poor infection control management in long term care (LTC) facilities at the beginning of the pandemic, linking ~ 80% of deaths in Canada to LTC (https://www.cihi.ca/sites/default/files/document/covid-19-rapid-response-long-term-care-snapshot-en.pdf). In contrast, woman in reproductive age from 20 to 49 years old has accounted for 24.6% (36,871/14,9851) of all COVID-19 infections in Canada, and from the same group 2.5% (914) were hospitalized, 0.5% (189) admitted to ICU, and the mortality rate has been 0.06% (23/36,871) (https://health-infobase.canada.ca/covid-19/epidemiological-summary-covid-19-cases.html#fn3).

Federal, provincial and local governments of all 10 provinces and 3 territories in Canada have cooperatively worked together in response to this pandemic. Measures imposed to fight the COVID-19 pandemic have affected the normal functioning of almost all aspects of daily and social life including closing of schools, universities, public offices and spaces, as well as cancellation of all non-essential activities including elective medical diagnostic and treatment procedures. At the beginning of the pandemic (mid March until mid May, 2020) many fertility clinics in Canada closed completely or provided only emergency care, as in many other countries. Implementation of mitigation strategies such as physical distancing, wearing masks, restriction of non urgent medical care, limiting social gatherings, minimization of non essential traveling, border closing, expanding the testing capacity and contact tracing had a positive effect on limiting the spread of COVID-19 and flattening the curve of infection incidence. Eventually this was followed by staggered and staged reopening of businesses in Canada. The ART (Assisted Reproductive Technologies) services in Canada were reopened during the first stage of easing the restrictions and returning back to normal functioning which included resumption of all non-essential medical treatment and diagnostic procedures [2], with adoption of changes in protocols to maximize the protection of patients and healthcare staff.

### Pathophysiology of SARS-CoV-2

Coronaviruses (CoVs) are round and enveloped, positive sense, single strained RNA viruses, ~ 30 kb in size, and ranging 65-125 nm in diameter [3]. They are classified in four genera: Alfa, Beta, Gama and Delta [4]. SARS-CoV-2 is a Beta CoV, part of subgroup 2B, with a crown-like appearance on its surface. Its genetic sequence has at least 70% homology with the SARS-CoV and ~ 50% with the Middle East Respiratory Syndrome (MERS) CoV [5].

SARS-CoV-2 consists of three structural proteins: 1) Spike (S), a transmembrane glycoprotein protruding from the virus surface, which determines the diversity of corona viruses and host tropism, with two subunits, a)S1, which is responsible for attachment to the host cell receptor; and b)S2 which is responsible for the fusion of the membranes of the virus and the cell [6]; 2) membrane (M), which determines the shape; and 3) envelope (E), a protein responsible for passage and assembly during viral morphogenesis [7]. The capsid of SARS-Cov-2 includes the RNA genome complexed with a nucleocapsid (N) protein that has three major regions: 1) N-terminal domain (NTD), responsible for RNA binding, 2) central linker domain, and 3) C-terminal (tail) domain (CTD), responsible for dimerization of N-proteins, which regulate replication, transcription and translation in the host cell [8].

The first step in the life cycle of the SARS-CoV-2 within the host is attachment to the host cell receptors, and consequently penetrating the cell through fusion with the host cell membrane (endocytosis). When the virus is intracellular, viral RNA enters the nucleus to replicate, and viral mRNA is utilized to make viral proteins (biosynthesis). The next steps are maturation of new viral particles, packaging in vesicles, transfer to the cell membrane, and release [7, 9].

Angiotensin converting enzyme 2 (ACE2) is a functional receptor on alveolar epithelial type 2 (AT2) cells and an entry point for the SARS-CoV-2 [10]. The spikes of SARS-CoV-2 (S-protein) have strong affinity for the ACE2 receptor, and after attachment, the viral genome and nucleocapsid are liberated into the host cell cytoplasm [11, 12]. SARS-CoV-2 needs TMPRSS2 (transmembrane, serin protease-2) to cleave the viral S-protein, and enable fusion between the viral and host cellular membrane [10, 13, 14]. The co-expression of both ACE2 and TMPRSS2 genes is necessary for infection to occur, since SARS-CoV-2 uses the ACE2 receptor for entry and the serine protease TMPRSS2 for S protein priming [10].

ACE2 expression is found in the heart (7.5% of myocardial cells), ileum (30%), kidney (4%), bladder (2.4%) and in the respiratory tract (~ 2%) [15, 16]. All tissues that have more than 1% expression of ACE2 receptors could be a target for the SARS CoV-2 [16].

### Transmission of SARS -CoV-2 and symptoms of COVID-19

SARS -Cov-2 infection can cause several sequelae, including: severe acute respiratory distress syndrome (ARDS), severe lower respiratory infections, coagulopathy, vascular disease, stroke in younger adults, neurological defects (loss of taste and smell), kidney disease, Kawasaki syndrome in young children, other multisystemic pathologic effects, and death. The spectrum of symptoms in COVID-19 infected patients ranges from asymptomatic to mild (flu-like symptoms) in 81% of the cases, severe in 14% (hospitalization and oxygen support), critical in 5% (mechanical ventilation), with a case fatality rate of 2.3–3% [17, 18]. Severity of the disease is related to the patient’s age and comorbidities. Patients over 60 years of age with co-morbidities such as diabetes and/or hypertension have the worst prognosis, with ~ 44.5% (95% CI 27–61.9) of this patient group experiencing a severe form of the disease [18]. A study assessing pregnant women admitted to a New York City hospital for delivery, randomly tested for COVID-19, showed that 15.7% (33/210) were positive during their admission, and only 20% (7/33) of the confirmed positive women had symptoms at admission or during their hospital stay [19].

SARS CoV-2 infection has a broad spectrum of symptoms such as fever 85.6% (95% CI 81.3–89.9%), cough 65.7% (95% CI 60.1–71.4%), tiredness 42.4% (95% CI 32.2–52.6%), shortness of breath 21.4% (95% CI 15.3–27.5%), dyspnea 18.6%, headache 13.6%, join or muscle pain 14.8%, olfactory disfunction 52.73% (95% CI 29.64–75.23%), gustatory dysfunction 43.93% (95% CI 20.46–68.95%), nausea and vomiting 5%, diarrhea 3.7%, and conjunctival congestion 0.8% [15, 18, 20].

SARS-CoV-2 is very contagious with a transmission rate of Ro (effective reproduction number) =2.2 (1.4–3.9) [21]. The mean incubation period of SARS CoV-2 is 5.2 days (2–14 days) with 95% of cases within 12.5 days (95% CI [5.3–19]) [21]. Transmission in most cases is through respiratory droplets (coughing, sneezing, talking) or by contact with contaminated physical objects. It has been shown that this virus can persist in aerosols up to 3 h, however, there is limited evidence on the infectious potential [22]. SARS-CoV-2 can persist on plastic and stainless steel up to 5 days with a significant reduction after 72 h [22]. On cardboard it does not persist more then 24 h, and on copper there was no presence of SARS-CoV-2 in 4 h [22, 23]. Asymptomatic carriers can be contagious as well [24, 25]. Chu DK et al. [26] systematically analyzed the effect of physical distancing, face masks and eye protection to prevent person to person transmission of SARS-CoV-2 and COVID-19. The best protection and mitigation of transmission of SARS-CoV-2 is physical distancing of at least 2 m, with estimated chance of transmission of 2.6% if the distance is at least 1 m, and 12.8% if the distance is less then 1 m. With further increase of the distance for each additional meter the relative protective effect increases 2.02 times. This study also showed that N95 respiratory masks or similar face masks in health care and non-health care settings are effective in prevention of transmission and infection with SARS-CoV-2; adjusted risk (aRR) = 3.1% with face mask vs. 17.4% without face mask. Eye protection could add additional benefit in reduction of transmission and infection with SARS-CoV-2, aRR of 5.5% with eye protection vs. 16% without [26].

## SARS-CoV-2 susceptibility of reproductive tissues

ACE2, the functional receptor for SARS-CoV-2, is a key component of the renin-angiotensin system (RAS), modulating the cleavage of angiotensin II (Ang II) and Ang (1–7) [27]. After cell invasion, COVID-19 disrupts the RAS system, by downregulating ACE2 expression in the host cells, leading to an increased proinflammatory response by Ang II [27, 28]. Ang II, ACE2 and Ang (1–7) regulate basic functions in the male and female reproductive systems. In the female, these include folliculogenesis, steroidogenesis, oocyte maturation, ovulation [29], and endometrial regeneration [30]. In the male, testicular ACE2 may regulate testicular function [31], plays a role in sperm function [32], and may be important for sperm’s contribution to embryo quality [33]. Since SARS-CoV-2 enters the cell by binding to the ACE2 receptor, reproductive cells and/or tissues expressing it are potentially vulnerable to the virus, and their functions may theoretically be disturbed.

ACE2 receptors are much more abundant in the male reproductive system than the female reproductive system. Low expression of ACE2 was demonstrated in the fallopian tube (ciliated and endothelial cells), ovary, vagina, cervix and endometrium [34–36]. On the other hand, ACE2 expression in the testis is among the highest observed, with high expression in Leydig and Sertoli cells and medium expression in glandular cells of the seminal vesicle [34, 37, 38]. As a result, it is expected that the testes will be more vulnerable than the ovaries to the detrimental effects of a SARS-CoV-2 infection.

### COVID-19 and gonadal pathology

Viruses such as hepatitis B, mumps, and HIV can enter the male reproductive tract and may impair fertility by causing orchitis [39]. During the past SARS epidemic, one study on testis specimens obtained from deceased SARS-CoV patients indicated that orchitis can be a complication of the virus [40]. Given the high genomic similarity between SARS-CoV and SARS-CoV-2 [41], the novel coronavirus may have the ability to cause the same testicular complications. A few papers reported on clinical manifestations of COVID-19 in the testes. A recent study of men with COVID-19 reported that almost one fifth (19%) of participants experienced scrotal discomfort, comparable to that with orchitis [42]. Four case reports described males with testicular pain as an atypical presenting symptom of COVID-19. Two cases described a benign course of SARS-CoV-2 infection in a 42 and 49-year-old man [43, 44]. The third one was a lethal case in a 43-year-old man [45]. Another case was a diagnosis of orchi-epididymitis in a 14-year-old boy with concurrent COVID-19 infection [46].

Data on pathological findings in gonadal tissues of COVID-19 patients are scarce. Yang et al. reported on pathological changes in 12 testes from deceased COVID-19 patients. Although no evidence for the virus was found in the testes in the majority (90%) of the cases by RT-PCR, 9 of 11 cases showed moderate or severe injury to Sertoli cells and seminiferous tubules. A significant reduction of Leydig cells was observed compared to controls, and mild inflammatory infiltrates in the interstitium were demonstrated [47]. Another study described histological findings in extrapulmonary organs in 10 fatal cases of COVID-19 in Brazil. Two testicles were examined, both demonstrating histological signs of orchitis [48]. In contrast, the autopsy results of 12 COVID-19 patients, showed no abnormalities in the gonads of either males (*n* = 9) or females (*n* = 3). However, in the same study 6 of the 7 prostates examined had microthrombi [49]. Overall, the little evidence that exists, suggests involvement of male reproductive tissues, but not involvement of female gonads. Further studies in both sexes are needed to confirm these findings.

### COVID-19 effect on reproductive hormones

One of the main functions of the ovaries and testes is steroidogenesis. Hence, assessment of sex hormone levels could provide an assessment of gonadal function in COVID-19 patients. The gonadal function in critically ill men is unknown, mainly because serum Testosterone (T) concentrations are not routinely measured in clinical practice [50].

Two studies assessed the impact of COVID-19 on male reproductive hormones. Ma et al. [51] compared sex-related hormone levels between 119 reproductive-aged men with SARS-CoV-2 infection and 273 age-matched controls. Most patients had moderately severe disease. A higher serum luteinizing hormone (LH) and a lower ratio of T to LH were observed in the COVID-19 group. Rastrelli et al. [52] investigated hormone levels in male patients admitted to the respiratory intensive care unit (ICU) with SARS-CoV-2. Worsening of clinical status was coupled with a progressive reduction in T levels and increase in LH levels [52]. However, these results should be interpreted with caution, since the sex hormone baseline in these patients before infection was not available. Furthermore, hypogonadism is a common finding in systemic illnesses. In the case of COVID-19, it is unknown yet whether the low T levels observed are the result of a direct effect by COVID-19 on gonadal function by a non-specific result of a severe systemic illness [50, 53]. Follow up and evaluation of reproductive function in recovering patients is required to assess the duration of these effects after recovery. Future studies should also focus on investigating possible underlying mechanisms [51].

In females, a severe acute illness may alter the hypothalamic-pituitary gonadal (HPG) axis function, decreasing the endogenous production of Estrogens and Progesterone [54]. To date, there are no published studies examining the effect of COVID-19 on sex-related hormone levels in female patients.

### COVID-19 and semen parameters

Aspects of the viral illness, such as fever, inflammation, and dysregulation of HPG axis, may also impair testosterone secretion and sperm production [55, 56]. Increased oxidative stress, as may be caused by COVID-19 [57], could reduce sperm motility and increase sperm DNA fragmentation [58–60].

Therefore, it is important to evaluate semen quality for a better understanding of the impact of COVID-19 on testicular function. A few studies evaluated semen characteristics in COVID-19 patients [51, 61, 62]. These studies assessed patients with mild and moderate disease severity. Ma et al. included 12 semen samples from reproductive-aged patients. The interval between semen collection and disease onset ranged from 56 to 109 days. Most patients (66.7%) had normal sperm parameters. Four patients with moderate disease had low sperm motility with higher sperm DFI; two of these four also had poor sperm morphology. Three patients performed a semen analysis before an infection with Covid-19. When current and previous samples were compared, one case showed a decrease in sperm motility (asthenospermia), the second showed no difference in sperm parameters, and the third patient’s parameters were also normal, but a decrease in total motile sperm number was observed post infection [51]. Another study reported a significant impairment of sperm parameters (concentration, motility) in subjects recovering from moderate disease. In this study, a shorter interval of 37–52 days between semen collection and disease onset was reported [61]. A third study showed contradicting results, as all samples (*n* = 23) from patients with mild (78%) and moderate (22%) disease had normal semen parameters. The median interval from diagnosis to providing the semen sample was 32 days in that study [62].

Overall, these preliminary data suggest that mild disease does not appear to have a negative effect on spermatogenesis. Yet, given the variable nature of semen quality, the small sample sizes, semen samples provided in intervals shorter than 3 months from the disease onset, and lack of long term follow up, makes it imperative that further studies are performed to clarify the effect of COVID-19 on spermatogenesis. Future studies should be statistically powered, also include men recovering from severe disease, and take into consideration possible confounding factors, such as medications used to treat the infection.

## Sexual transmission of COVID-19

The male reproductive system may be contaminated by viruses during viremia, due to compromised blood–testes/vas deferens/epididymis barriers, especially in the presence of systemic or local inflammation. Salam et al. [63] reported the presence of 27 viruses, possibly resulting from viremia, in human semen. For many of them, data regarding sexual transmissibility is lacking [63]. Infectivity, which is a prerequisite for viral transmission, depends on the infectious dose and the exposure route. Nowadays, virus detection is largely achieved by means of molecular methods, which have replaced standard procedures of virus isolation. However, the only definitive way to prove infectivity is isolation of the virus. The fact that sexual transmission can rarely be confirmed for some viruses, despite the detection of RNA in the semen long after the acute infection, emphasizes the shortcomings of molecular detection alone in understanding transmissibility [64].

Should sexual contact be a matter of concern in the case of COVID-19? The need for cautious sexual behavior is clear, since salivary viral shedding has been reported in COVID-19 patients for up to 11 days after hospitalization [65]. Some may claim it is unnecessary to investigate genital secretions in the context of sexual transmission, as air droplets and human contact are the main routes of this virus spread, and both will most likely happen during sexual activity anyhow [66]. However, research shows that sexual transmission may possibly cause delayed outbreaks after the first wave of cases, and the absence of a virus in the genital secretions should not be assumed for traditionally non–sexually transmitted viruses [63, 64]. Furthermore, transmission via semen is especially relevant in infertile patients undergoing ART, due to the theoretic possibility of direct transmission via intracytoplasmic sperm injection (ICSI). It is possible that this virus could affect early embryogenesis, and this should be taken into consideration in future research [67].

### Sexual transmission in men

Several studies have attempted to investigate the presence of COVID-19 in semen of infected or recovering men, showing conflicting results (Table 1) [42, 61, 62, 68, 69, 71, 72]. Most of these studies suggested it is highly unlikely that the COVID-19 can be sexually transmitted by men. Song et al. [68] examined semen samples of 12 recovering men and one testis tissue sample from a deceased patient. Both the semen and testis specimen were negative for SARS-CoV-2 RNA [68]. Guo et al. [62] reported no detection of SARS-CoV-2 RNA in semen samples of 23 patients with a recent infection or recovering from COVID-19. The median interval from the diagnosis to providing the sample was 32 days [62]. Similar results were reported by Pan et al. in 34 recovering male patients [42]. In another study, no viral RNA was detected in semen samples obtained from recovered men (*n* = 18) 8–54 days after absence of symptoms, nor from 2 patients with acute COVID-19 infection [61]. A different study included semen samples from 16 hospitalized male patients taken during the acute stage of disease, and all tested negative for SARS-CoV-2 PCR [69]. Zhang et al. examined expressed prostatic secretion (EPS) in 10 male patients with confirmed COVID-19 (3 positive, 7 recovering). None of the patients had positive SARS-CoV-2 RNA in EPS [70]. Only one study demonstrated that SARS-CoV-2 can be present and detected in the semen samples. 26.7% (4/15) of the patients who were in the acute stage of infection (6–11 days from onset of symptoms) and 8.7% of the patients (2/23) who were recovering (2–3 days after achieving clinical recovery) had positive test results for SARS-CoV-2 in semen [71]. However, there is still no definitive data to prove that spermatozoa serve as vectors for sexual transmission of COVID-19. The conflicting findings regarding the presence of this virus in semen are based on studies with small sample sizes with proven COVID-19 infection, in both acute and recovery phase of the disease with limited data on severity of the disease, the methods for sample collection and contamination control. In addition, the sensitivity and specificity of the RT-PCR methods used to detect SARS-CoV-2 in the semen were not reported. Overall, evidence suggests low detection (3.9%, 6/152) of the virus in semen. However, the possibility of SARS-CoV-2 presence in semen of men who unknowingly have the virus cannot be ruled out. This necessitates the need for further studies in symptomatic and asymptomatic men before any final conclusions can be drawn.

**Table 1:** Summary of studies investigating the presence of SARS-CoV-2 in semen samples

Study	Disease stage	No. of semen samples		Interval from diagnosis/ test sample	SARS CoV-2 (−) RT PCR samples	SARS CoV-2 (+) RT PCR samples
Song et al. [68]	Recovering man	12	1 Testes^a^	N/A	100% (13/13)	0
Guo et al. [62]	Recent/recovering	23		Median 32 days	100% (23/23)	0
Pan et al. [42]	Recovering	34		Median 31 days	100% (34/34)	0
Kayaaslan et al. [69]	Hospitalized patients in acute stage	16		Median 1 day	100% (16/16)	0
Holtman et al. [61] Cohort study	16 recovered/2 acute phase of infection	18		8–54 days after absence of symptoms	100% (18/18)	0
Zhang et al. [70]	3 positive/7 recovering	10 expressed prostatic secretion		< 3 days after positive test, 1 day after SARS Cov-2 (−) test	100% 10/10	0
Li D et al. [71]	15 acute phase of infection + 23 recovered	38		Acute phase 6–11 days from symptoms onset, 2–3 days after clinical recovery	84% (32/38) (13/15 + 19/23)	16% (6/38) (4/15 + 2/23)

^a^(from a deceased patient)

### Sexual transmission in women

Three studies examined female genital tract secretions for the presence of SARS-CoV-2, showing consistent results [73–75]. Aslan et al. [73] included 12 pregnant women with confirmed COVID-19 and moderate symptoms. Vaginal swabs were obtained during the hospitalization period. All lower genital tract samples were negative for SARS-CoV-2 [73]. Qiu et al. analyzed vaginal samples of 10 women with severe COVID-19 pneumonia admitted to an ICU. All samples tested negative for the virus [74]. The third study by Cui et al. [75] included 35 both reproductive-age and postmenopausal women with mild to moderate SARS-CoV-2 disease. The interval from the first symptoms of COVID-19 to the time of taking the samples was 8–41 days. All samples from the lower genital tract (including vaginal fluid and cervical exfoliated cells) were negative for SARS-CoV-2 [75]. In addition, a systematic review of case series and case reports among other parameters showed no presence of SARS-CoV-2 in vaginal mucosa and breast milk in all 28 tested pregnant women [76].

Although these studies consisted of small numbers of patients, they included women in various age ranges and varying degrees of disease severity. Currently, the available evidence suggests that the female genital tract is unlikely a route of SARS-CoV-2 transmission.

## Effects of COVID-19 during pregnancy: fetal, maternal and neonatal risks

COVID-19 infection has raised concerns for pregnant woman and their fetus due to physiological changes in immunity during pregnancy, maternal susceptibility to respiratory infections, increased oxygen requirements, and risks associated with treatment during pregnancy.

The fatality rate in pregnancy for previous pandemics, including: the 1918 Spanish influenza pandemic, the SARS-CoV pandemic, and the MERS pandemics were ~ 27–50%, 25–30%, and ~ 40% respectively [77]. Knowledge regarding the effects of COVID-19 on pregnancy, including vertical transmission and perinatal infection, is based on very limited data. A systematic review and meta-analysis of COVID-19 in pregnant women from 77 cohort studies by Allotey et al. [78], showed an overall 10% (95% CI [7–14%]; 28 studies, 11,432 women) COVID-19 positivity in pregnant and recently pregnant women attending or admitted to hospital for any reason. This relatively high incidence may partially reflect the increased vigilance and screening for COVID-19 in pregnant women admitted to hospitals, and is in line with admissions in regions that were mainly reporting data from the peak epidemiological curve of large outbreaks [78].

COVID-19 disease has a similar spectrum of symptoms in pregnant woman as in non-pregnant woman, such as: fever ~ 40%, cough 39%, shortness of breath 13.2%, malaise 13%, muscle pain 10%, diarrhea 3.7–7%, sore throat 3.4%, headache 40%, chills 28%, loss of taste and smell ~ 16%. However, most of the symptoms are less frequent during pregnancy [76, 78–80]. Pregnant women with COVID-19 are less likely to report symptoms of fever (OR 0.43, 95% CI [0.22 to 0.85]; I2 = 74%; 5 studies; 80,521 women) and myalgia (0.48, [0.45–0.51]; I2 = 0%; 3 studies; 80,409 women), but they are more likely to need admission to an ICU (OR 1.62 [1.33–1.96]; I2 = 0%) and require invasive ventilation (1.88, [1.36–2.60]; I2 = 0%; 4 studies, 91,606 women), compared with non-pregnant women of reproductive age [78].

The overall data from 26 studies, that included 11,580 women, showed 73 deaths in COVID-19 positive pregnant women (0.1, 95% CI [0.0–0.7%]) [78]. The severe form of COVID-19 manifested in 13% of pregnant women with suspected or confirmed COVID-19 infection (6–21%; 21 studies, 2271 women); admission to an intensive care unit was required for 4% (2 to 7%; 17 studies, 10,901 women), 3% (1 to 5%; 13 studies, 10,713 women) required invasive ventilation, and 0.4% (0.1 to 0.9%; 9 studies, 1935 women) required extracorporeal membrane oxygenation [78]. Factors associated with severity were pre-existing maternal comorbidities, such as increased maternal age (1.78, 1.25 to 2.55; I2 = 9%; 4 studies; 1058 women), high body mass index (2.38, 1.67 to 3.39; I2 = 0%; 3 studies; 877 women), chronic hypertension (2.0, 1.14 to 3.48; I2 = 0%; 2 studies; 858 women), and pre-existing diabetes (2.51, 1.31 to 4.80; I2 = 12%; 2 studies; 858 women).

Vertical transmission happens if an infected pregnant woman transmits the infection to her fetus/infant during the fetal, intra-partum or post-partum period. The route of vertical transmission can occur through the placenta in-utero, maternal - neonatal contact during delivery, or during breastfeeding. ACE2 receptors necessary for viral infection are expressed in the placenta (syncytiotrophoblast, cytotrophoblast, endothelium and vascular smooth muscle from both secondary and primary villi), ovaries, uterus and vagina, and may be involved in vertical transmission [81, 82]. A transcriptomic study on single cell expression profiling has shown that ACE2 and TMPRSS2 positive cells are present in human trophectoderm and placenta throughout pregnancy, indicating susceptibility for SARS-CoV-2 infection of these tissues and the possibility of intrauterine fetal infection [83]. In addition, there are other potential SARS–CoV-2 receptors, such as BSG (CD147) and NRP1(enriched in trophoblast) receptors (expressed in the pre-gastrulation embryo), and proteases, such as CTLS (Cathepsine L-like cysteine protease), a key protease in embryos, suggesting there are other routes for infection of embryos that need further investigation [84–87].

Kotlyar A et al. [88] in their systematic review and meta-analysis of 38 cohort/case series (total *n* = 936) reporting on neonatal infection immediately after or within 48 h after birth, in COVID-19 positive pregnant women, showed a pooled proportion of 3.2% (95% CI 2.2–4.3%) for vertical transmission of COVID-19 infection [88].

Allotey et al. [78] reported a spontaneous preterm birth rate of 6% (95% CI [3–9%]; I2 = 55%; 10 studies; 870 women) in women with COVID-19. The odds of any preterm birth were higher in pregnant women with COVID-19 compared with those without the disease (OR = 3.01, 95% CI [1.16–7.85]; I2 = 1%; 2 studies; 339 women). A quarter of all neonates born to mothers with COVID-19 were admitted to the neonatal ICU, revealing an increased risk of admission, compared to those born to mothers without COVID-19 (OR = 3.13, 95% CI [2.05–4.78], 1 study, 1121 neonates) [78].

The overall rate of caesarean section (CS) in COVID-19 mothers is high, ranging from 67.2 to 94%, and vaginal delivery from 6 to 32.8% of cases [80, 88–91]. Most of these women were in their 3rd trimester when the infection occurred.

Outcomes of neonates born from SARS CoV-2 positive mothers during pregnancy was systematically summarized by Yoon et al. [90]. They included 16 case series and 12 case reports, reporting overall on 223 neonatal outcomes [90]. In total, 201 delivered live born infants before the end of the study, with gestational age at birth ranging from 30 wks + 6 days to 41 wks + 5 days, and birth weight ranging from 1880 to 4050 g. SARS CoV-2 detection within 36–48 h after birth showed positive results in 2.4% (4/167) of tested neonates. All four SARS CoV-2 positive neonates were delivered by CS due to maternal Covid-19 pneumonia and all manifested signs of pneumonia on chest imaging, all had a negative RT-PCR nasopharyngeal (NP) swab 1 week after birth, and all recovered well [90]. The authors reported a group of four neonates with false positive tests, one of them had a positive SARS-CoV-2 PCR test from NP swab, but it was negative when repeated, and other three neonates showed positive SARS CoV-2 IgM, but their NP samples were negative as well. In that systematic review the incidence of premature birth was 25.9% (48/185), small for gestational age (SGA) 8.3% (5/60), low birth weight (LBW) 15.6% (15/96) and neonatal asphyxia was reported in 1.8% (3/168). Overall, 93% (68/73) of the infants born to COVID-19 positive mothers were asymptomatic. Only one (1/177, 0.6%) newborn born at 34 wks + 5 days died due to gastric bleeding (disseminated intravascular coagulation) and multiple organ failure 9 days after birth. His nucleic test for SARS Cov-2 was negative before his death. That study reported a frequency of preterm rupture of membranes (PROM) of 12.7% (16/126) of cases, and fetal distress in 10.6% (15/141). Five mothers received mechanical ventilation in ICU and two (2/5) developed acute respiratory distress syndrome and delivered stillbirths [90, 92].

Pathohistological changes in placentas from COVID-19 positive pregnant women commonly showed vascular malperfusion with central and peripheral villous infarctions, fibrin deposition and chorionic villitis or intervillositis with inflammatory infiltrate of CD68+ macrophages and T cells [93–95]. In a series of cases, RT-PCR testing of placentas from SARS CoV-2 positive pregnant women showed that 20% (4/20) were positive for SARS CoV-2 [88]. This is evidence that SARS CoV-2 can infect the placenta, however transmission to the fetus was not detected in 2/4 fetuses from SARS CoV-2 positive placentas. Both were diagnosed in the second trimester of pregnancy, 19 and 22 wks respectively, and both pregnancies ended with fetal demise [88, 94, 96]. There was an additional reported case, where a woman who delivered at 29 wks due to disease severity, had visible virions in the syncitiotrophoblast on electron microscopy, but the fetus was negative for SARS CoV-2 [97].

Some studies have shown a linkage of fetal vertical transmission with maternal disease severity. Penfield et al. [98] showed that 3 of 11 placental or amniotic membranes swabs of SARS CoV-2 positive mothers, after CS delivery were positive. The three positive placental cases were from mothers in severe/critical stage of the disease, whereas the other 7/8 placentas were negative with mild disease and delivered vaginally, and only one of the SARS CoV-2 negative patients (1/8) was in the critical phase of the disease and delivered by CS. All newborns 11/11 tested negative for SARS Cov-2 and did not have COVID-19 symptoms at the first or fifth day of life, which raises the question that swabs from the placenta and membranes might be mixed with maternal tissue, amniotic fluid and/or maternal blood [98]. Furthermore, Chen et al. [99] described nine pregnant women with mild symptoms and laboratory confirmed SARS CoV-2, who had a live birth by CS in the third trimester of pregnancy. None of the mothers had severe COVID-19 symptoms, pneumonia or died. All six, tested, cases of infant/mother pairs had negative SARS CoV-2 swabs/samples from multiple sites (amniotic fluid, cord blood, neonatal throat swab and breast milk) [99].

Yan J. et al. [89] analyzed 116 pregnant women (65 laboratory confirmed SARS CoV-2 and 51 clinically diagnosed with COVID-19 pneumonia) admitted to hospital. 23.3% (27/116) cases were asymptomatic of the disease, however after close evaluation due to positive epidemiological history and close contact with confirmed COVID-19 positive patients 21/27 were clinically diagnosed with pneumonia. The study showed that only 6.9% (8/116) of positive pneumonia maternal cases during pregnancy progressed to severe pneumonia and admitted to ICU without maternal death [89]. In this study, 8 patients presented with COVID-19 during the first and early second trimester, and 1/8 (12.5%) miscarried. The rate of preterm birth (< 37 GW) was 21.2% (21/99), 26.8% (6/21) of which involved PROM [89, 100]. The most common symptoms in COVID-19 positive mothers during the second and third trimester were fever (42.3%) and cough (31.8%). There were laboratory findings of leucocytosis in 31.5%, lymphocytopenia in 43%, increased C-reactive protein in 63%, and patchy shadowing or ground glass opacity in the lungs in 96% [89, 90]. The study reported that 47% (47/99) of the neonates were transferred in neonatal ICU, neonatal death in 1% (1/99), 76.7% (76/99) were discharged, 23.2% (23/99) were still in hospital. Importantly, all 86 neonates were negative for SARS-CoV-2 [89].

Juan J. et al. [76], in their systematic review analysed 24 studies (9 case series and 15 case reports), involving a total of 324 women diagnosed with SARS CoV-2. Gestational age at admission ranged from 5 to 41 weeks, and at delivery 28–41 wks. There were 4 women who miscarried [76].

Huntley et al. analyzed 13 studies, involving a total of 462 pregnant patients with a laboratory confirmed COVID-19 diagnosis. Maternal ICU admission rate was 3% (8/263), 1.4% (3/209) had critical disease, no (0/313) maternal deaths were reported, preterm birth rate was 20.1% (51/284) (iatrogenic and spontaneous), and the neonatal death rate was only 0.3% (1/313). There was no vertical transmission detected in all 310 neonates [101].

COVID-19 symptom progression during pregnancy compared to non-pregnant women was analyzed by Hsu at al., who showed that 8.3% (36/431) of the COVID-19 patients during pregnancy had severe disease, of whom 86% (31/36) progressed to a critical stage and were admitted to the ICU [102]. Another study by Blitz et al. [103], reported no significant difference between the rates of ICU admission in hospitalized COVID-19 pregnant woman (9.8%; 8/82), and non-pregnant COVID-19 positive patients (15.1%; 50/3320) (*p* = 0.22). These studies suggest that hospitalized pregnant SARS CoV-2 positive patients are not at an increased risk for severe disease progression compared with non-pregnant COVID-19 positive hospitalized woman [102, 103].

In contrast, Ellington et al. [79] reported that COVID-19 positive pregnant woman have higher chance to be hospitalized vs. non pregnant COVID-19 positive woman (31.5% vs. 5.8% respectively). However, they did not stratify for the underlying causes of hospitalization in this cohort (ie delivery, pregnancy related procedures, or Covid-19 related complications). The authors reported an adjusted risk ratio (aRR) of 1.5 (CI 1.2–2.4) for pregnant COVID-19 positive patients to be admitted to the ICU, and an aRR of 1.7 (CI = 1.2–2.4) to receive mechanical ventilation. The fatality rate was similar between pregnant COVID-19 patients and non-pregnant woman, 16/8207 (0.2%) and 208/83205 (0.25%) respectively (aRR = 0.9, 95% CI = 0.5–1.5). In this study, the pregnant woman cohort had more comorbidities overall vs. non-pregnant woman included: chronic lung disease, 21.8% vs 10.3%; diabetes mellitus, 15.3% vs. 6.4%; and cardiovascular disease, 14% vs 7.1%, respectively [79]. Collin et al. reported that the incidence of COVID-19 and admission to ICU for pregnant/post-partum woman in Sweden was 14.4 per 100,000 (CI 7.3–23.4) compared to 2.5 per 100,000 (CI 1.8–3.5) for non-pregnant COVID-19 positive woman, RR = 5.39 (CI 2.89–10.08); and a higher risk of requiring mechanical ventilation for admitted pregnant/post-partum woman, RR = 4 (CI 1.75–9.14) [104]. These results warrant careful and close follow up of all COVID-19 positive pregnant and postpartum women until more data is available. It should be noted that these studies potentially capture differences in health care systems and local management of epidemics.

In Ontario, Canada, the BORN (Better Outcomes Registry and Network) database, during the period from March, 1st to May, 29th, 2020 captured cases of COVID-19 pregnant woman from 57.4% (54/94) of the hospitals and 31.5% (29/92) of the midwifery practice groups in their network [105]. There were 36 reported pregnant COVID-19 patients in Ontario for this period; 75% (27/36) confirmed and 25% (9/36) suspected. The most common symptoms in confirmed cases were: cough (44.4%), fever (40.7%), anosmia (22.2%), myalgia (14.8%), malaise (14.8%), shortness of breath (14.8%), headache (11.1%), anorexia (11.1%), rhinitis (7.4%), sore throat, diarrhea, vomiting, loss of taste, and chest pain; 25.9% (7/27) of the cases were asymptomatic. Three of confirmed cases 11.1% (3/27) had complications (2 patients had pneumonia/abnormal X-ray finding, and 1 patient had coagulopathy). Infection was confirmed at different gestational ages; 4 cases at < 20 wks, 3 cases at 21–32 wks, 2 cases at 33–36 wks, 13 cases at 37–40 wks, and 5 cases at > 40 wks. Birth outcomes available to date are as follow: 21/ 27 live birth, 2/27 data is missing, and 4/27 were still pregnant. Four of 21 had preterm births, and 17 of 21 had term/post-term births. There was one (1/12) infant who tested positive for SARS CoV-2 after birth (https://www.bornontario.ca/en/data/resources/Documents/2020-06-04-BORN-Ontario%2D%2D-COVID-19-in-Pregnancy-in-Ontario-Report%2D%2DFinal.pdf).

## Fertility guidelines during COVID-19 pandemic

In response to the COVID-19 pandemic, scientific and professional fertility societies around the world, such as American Society of Reproductive Medicine (ASRM), Canadian Fertility and Andrology Society (CFAS), European Society for Human reproduction and embryology (ESHRE) and the International Federation for Fertility Societies (IFFS) issued guidelines for couples who are undergoing or will undergo treatments involving ART [106–109]. All of them had in place COVID-19 working groups to monitor the evolution of this pandemic with updated scientific evidence on COVID-19 infection and current practice guidelines in line with local governmental and public health recommendations, as well as, provide continuous support for medical professionals and patients.

Implementation of restrictions in ART and fertility care worldwide at the beginning of the pandemic was to support the overwhelmed healthcare systems, and reduce over usage of personal protective equipment (PPE), which was in deficit worldwide. Although lacking scientific evidence on the risks associated with COVID-19 in pregnancy and patients undergoing ART, almost all guidelines issued by afore mentioned societies, shortly after WHO declared COVID-19 a global pandemic in mid March, 2020, recommended suspension of new fertility treatments – ovulation induction, intrauterine insemination and in-vitro fertilization, and all non-urgent gamete cryopreservation, as well as postponement of all embryo transfers and all elective surgeries and non-urgent diagnostic procedures. The reasons stated not to start new ART cycles were: 1) to avoid complications from ART and ART-pregnancies, 2) to avoid potential SARS-CoV-2 related complications during pregnancy, 3) to mitigate the potential, but unknown, risk of vertical transmission in SARS-CoV-2 positive patients, 4) to support the necessary re-allocation of healthcare resources, and 5) to observe the recommendations for social distancing. The recommendations directed couples using ART to discuss their treatment in detail with their treating physicians, and consider gamete cryopreservation or embryo preservation for those in-cycle to postpone embryo transfer. Only patients who required urgent stimulation and gamete preservation, such as oncology fertility preservation patients, were exempted from the restrictions and their medical care was not altered. All societies emphasized the importance of developing emergency preparedness plans for fertility clinics, monitoring of PPE supplies, active screening of patients and staff for COVID-19, increased utilization of telemedicine, and reduced clinic visits.

The eventual resumption of ART and fertility care worldwide was in line with the decline phase of local epidemiologic curves of incidence, as well as, a reduction of the impact of COVID-19 on healthcare resources [2, 110–113]. In Canada, the guidance on reopening of fertility clinics was given by the local provincial reopening strategies and outlined in the CFAS COVID-19 Update # 5 from April 29th, 2020, which was supported by two guiding CFAS documents “Guiding Principles to assist Canadian ART clinics to resume services and care” [114] and “Recommendations related to IVF Laboratory shutdowns and start-up during a Pandemic” [115] to assist clinics in their preparations for resumption of care. General principles include: a) Reduced face to face interactions to support social/physical distancing by virtual consultations; b) Pre-screening of all patients within 48-h of their intended appointment; c) Mandatory screening of all people entering the clinic; d) Informing patients of potential risks of treatment and/or pregnancy related to COVID-19; e) Changes in clinic policies because of COVID-19 as well as providing patients with local Public Health contact information; f) Continue fertility treatment if patient and staff safety is maintained with the recommendation that “patients with positive COVID-19 testing may require discontinuation of treatment (if safe to do so) until they have completely recovered, or until 4 weeks after onset, whichever is longer”; g) Defer treatment when necessary, especially patients under investigation for COVID-19, or who have had exposure to SARS CoV-2; h) Mandatory daily screening of all staff for symptoms and risk factors for COVID-19; i) Prevent infection and have control measures in place, in addition to physical distancing, that includes wearing appropriate PPE for the procedure as well as enhanced cleaning and disinfection protocols, consistent with current Public Health recommendations [114]. Recommendations related to laboratory operations during the current pandemic guided safe laboratory shutdown by properly managing the supply of medical gases and liquid nitrogen with an accent on cryo-storage safety (https://cfas.ca/_Library/_documents/CFAS-Guidance-Document-on-Cryo-Storage-June-2018.pdf [116]), proper shut down of incubators and other laboratory equipment, and managing supplies and ongoing maintenance ensuring enough consumables (culture media and vit/warm kits) for 2 months of full operation at all times [115]. The guidance on safe restart of ART laboratories indicated implementation of the necessary steps for the restart of equipment by following operational QC and maintenance protocols, external QC validation and thorough cleaning before clinical use, re-stocking of supplies. It also included elaborate directions on mandatory staff screening, strategies to provide safe workplace for staff and patients by scheduling, shifts, staggered restart of clinical procedures, extending the working day, limiting contacts between ART lab staff and other staff in the clinic, reducing number of deliveries, decontamination protocols in place for items received, limiting contact with delivery personnel, reducing number of shipments of biopsied cells to reference labs, proper use of PPE, and measures for infection containment by all staff [115].

### COVID-19 impact on ART in Canada

Yearly, there are ~ 1.5 million IVF cycles worldwide, resulting in ~ 400,000 newborns and about 0.3% of the overall live birth rate is from ART conceived babies [117]. In Canada during 2018, about 36,000 ART treatments were initiated, which resulted in 9581 clinical pregnancies (clinical intrauterine, heterotopic or ectopic), and 8631 ongoing clinical pregnancies (https://cfas.ca/_Library/CARTR/CFAS_CARTR_Plus_presentation_plenary_slides_FINAL_for_website_-_opened.pdf). There has been a tremendous increase in preimplantation genetics testing (PGT) over the years that has reached 6090 cycles using PGT for aneuploidy (PGT-A) and 640 cycles using PGT for monogenetic disorders (PGT-M), as compared to 2013, when Canadian clinics had 243 PGT-A cycles, and 240 PGT-M cycles (https://cfas.ca/_Library/CARTR/CFAS_CARTR_Plus_presentation_plenary_slides_FINAL_for_website_-_opened.pdf). The overall clinical pregnancy rate per embryo transfer (ET) cycle was 36.1%, with much lower rates for patients 41–42 years old (19.1%), and over 43 (11.6%) (https://cfas.ca/_Library/CARTR/CFAS_CARTR_Plus_presentation_plenary_slides_FINAL_for_website_-_opened.pdf).

At the beginning of the pandemic, closure of IVF centres significantly contributed to the dramatically decreased IVF procedures performed worldwide, and consequently decreased pregnancies and births in that period. The overall impact of COVID-19, measured in precise numbers, will likely be available in 1 year from now. Therefore, estimates are only based on available ART statistics. In Canada, 62.6% of all ART patients are over 35 years of age, 18.1% are over 41 years of age and these patients experience a relatively rapid decrease in their fertility potential, with increased chances of aneuploidy in their embryos, and an overall reduced chance of success for each fertility treatment cycle initiated (https://cfas.ca/_Library/CARTR/CFAS_CARTR_Plus_presentation_plenary_slides_FINAL_for_website_-_opened.pdf). ART cycles in patients less than 35 years old resulted in a live births rate of 41.5%; whereas for patients 40–43 years old it was < 5%; and only ~ 1% for women over 43 years of age [118]. Delaying fertility treatment in patients during the exponential decline of fertility due to the age related risk for embryo aneuploidy, has similar devastating consequences as for woman in need of urgent fertility treatment, such as those who are struggling with malignant diseases, autoimmune disorders, hematologic disorders and who need to be treated with gonadotoxic treatment, are candidates for urgent fertility preservation [119], as well as in women less than 35 years old, with significantly diminished ovarian reserve [120].

The contagiousness of the SARS CoV-2, has given rise to questions regarding the risk of transmission to, and between, human embryos, gametes, and reproductive tissues in cryo-storage within ART laboratories. A very low potential risk for cross contamination of SARS-CoV-2 in the IVF labs exists despite air control systems and negative pressure. This infection can be spread in different ways. Regarding human to human transmission, it is estimated that microorganism emission rates are 3.7 × 10^7^ bacterial and 7.3 × 10^6^ fungal genome copies per hour, per human, in indoor classrooms [121]. Furthermore, SARS-CoV-2 can persist in the air for more than 30 min when the temperature is above 22 °C. However, there have been no documented cases of disease transmission in a patient or recipient of donor reproductive tissues during IVF laboratory treatment (ie culture of embryos, cryopreservation, or storage). Therefore, it appears there is very low probability of SARS-CoV-2 affecting embryos or their recipients.

## Impact of COVID-19 on the desire for parenthood

One Italian study evaluated the impact of the COVID-19 pandemic on the desire for parenthood in couples of reproductive ages. This study included 944 women and 538 men aged 18–46 years, in heterosexual stable relationships. Interestingly, more than a third (37.3%) of participants who were planning to have a child before the pandemic, decided to abandon their intention during the quarantine, due to worries regarding future economic difficulties (58%) and/or potential risks to a pregnancy (58%). Of those who did not intend to conceive before the pandemic, 11.5% revealed a new desire for parenthood during quarantine, related to wanting a life change (50%) and a need for positivity (40%). However, only 4.3% of them actually tried to conceive [122]. Another study assessed the willingness to go ahead with the desire for pregnancy in infertile women during the outbreak. Almost half of the women (44.6%, *n* = 45) replied they would consider postponing their pregnancy plan due to COVID-19 [123].

## Psycho-social aspects

More than a third (36%) of infertile women, in general, will experience anxiety symptoms, affecting their quality of life [124]. Anxiety disorders are also prevalent in pregnant women. Some of them, such as panic disorder, have a higher prevalence during pregnancy than the lifetime prevalence rates for women in the general population [125]. During pandemics, the prevalence of psychological distress and symptoms of mental illness tends to be higher compared to routine periods [126]. A recent systematic review and meta-analysis reported that stress was the most common psychological after-effect among the general population during COVID-19 pandemic. It has been suggested that the reason for such a high burden is the prolonged quarantine [126]. Among the major stressors contributing to worldwide emotional distress and increased risk for psychiatric illness associated with the COVID-19 pandemic, are uncertainty, lack of resources, financial losses, violation of personal liberty, and conflicting messages from authorities [127]. Because they are coping with an additional burden, it would be expected that the infertile and pregnant subpopulations may be more prone to negative psychosocial effects. Several studies have assessed those aspects in these specific populations during the current pandemic.

### Perceptions, coping, emotions, and stress levels in infertile patients

To date, three studies assessing emotions and coping of infertile patients during the COVID-19 pandemic have been published. The first study included infertile females (*n* = 2202) in the USA, who were asked to rate their three top stressors from a list of 10 commonly reported life stressors, at three different time-points: January, early March, and April 2020. Only 6% of responders stated that infertility treatment, including IVF, should not be offered during the pandemic. Infertility was noted to be the most frequently reported top stressor at all three time-points (81.1, 69.3, 66.4%, respectively). Coronavirus was the third (53.6%) most common stressor in March but the second (63%) most common in April, almost as high-ranking as that of infertility itself. They concluded that despite the global pandemic, the stress of infertility remained a significant stressor, comparable to the pandemic itself [128].

The second study evaluated cognitive appraisals, emotions, and coping ability of patients whose fertility treatments were affected during lockdown in April 2020. Four men and 446 women (75% UK residents) completed the survey, and most of them (81%) had fertility tests or treatments postponed. Although the participants understood clinic closure was precautionary due to the unknown effects of COVID-19, some expressed anger and resentment at the unfairness of the situation and reported more negative than positive emotions (*p* < .001). Almost all participants reported stress, worry and frustration. The majority reported a slight to moderate ability to cope with closure, but 11.9% were not able to cope at all, reporting intense feelings of hopelessness, deteriorating wellbeing, and impaired mental health [129].

A recent study reported that 86% of the infertile women whose ART cycles were postponed due to the pandemic (*n* = 101) felt anxiety due to the possibility that their chances of achieving a pregnancy could be negatively affected by the delay. The state-anxiety levels were significantly higher in women older than 35 years. Diminished ovarian reserve and high duration of infertility were significantly associated with higher anxiety levels [123].

### Perceptions, coping, emotions, and stress levels in pregnant patients

Most studies assessing pregnant women in different countries during the COVID-19 pandemic, reported higher maternal anxiety and depression. Corbett at al. assessed maternal anxiety in 71 pregnant women during the delay phase of the pandemic in Ireland. Most women (83%) were not previously concerned about their own health, but during the delay phase over half of them (50.7%) worried about their health more often, or all the time. Women were more concerned about older relatives (83%), than their children (66%), and their unborn child (63%) [130]. An Israeli study explored the psychological distress in Jewish and Arab pregnant women (*n* = 336) during lockdown. Their results indicated that levels of all aspects of COVID-19-related anxiety were quite high (‘much’ or ‘very much’). Arab women were more anxious about each of the issues than Jewish women, emphasizing the potential vulnerability of subgroups, such as cultural minorities [131]. Another study assessed 946 Columbian pregnant women during the mitigation phase of COVID-19. The rate of psychological consequences of the pandemic was high, with half of the entire cohort reporting symptoms of anxiety and insomnia, and 25% with depressive symptoms [132]. A preliminary study investigated the effects of the pandemic on depression and anxiety in 260 pregnant women, without a history of psychiatric disorders. More than a third (35.4%) of participants had scores indicating they are considered at risk of developing postpartum depression. A significant correlation was found between the anxiety and the depression scores [133]. An Italian study evaluated pregnant women using the State–trait anxiety inventory. Their findings showed that the outbreak, and the subsequent lockdown, induced a significant increase in maternal anxiety as expressed by doubling of the number of women who reached an abnormal level of anxiety [134].

Only one study tried to determine the extent that COVID-19 aggravates prenatal distress and psychiatric symptomatology, by comparing pregnant women evaluated before versus after the pandemic. Women from the COVID-19 cohort (*n* = 1258) were almost twice (OR = 1.94, *p* = .002) as likely to present with clinically significant levels of depressive and anxiety symptoms, compared with the pre-COVID-19 group (*n* = 496) [135].

A French study is the first published that assessed the anxiety of women who conceived via ART during the COVID-19 pandemic. Interestingly, the majority (86.4%) of patients with ongoing pregnancies (*n* = 88) were psychologically able to cope with lockdown, reporting experience of only mild anxiety or no anxiety at all [136].

Data regarding the mental health of pregnant women who tested positive for COVID-19 is limited. Only one report including pilot data of 11 pregnant women is available. Their data demonstrated that even during maximal maternal anxiety, at the height of the pandemic, deaths were low. Depression scores followed a similar pattern. Lower scores were attributed to increased available information and reassurance [137].

## COVID-19 research related to fertility and reproduction in Canada

The Government of Canada has been providing funding for developing and implementing measures for rapid testing, management and reduction of transmission of COVID-19 through the Canadian Institute of Health Research (CIHR) (165,663,556 CAD) and provincial partners, such as the International Development Research Centre (IDRC) (6,554,078 CAD), in partnership with Partnership with Alberta Innovates (AI) 100,000 CAD, Michael Smith Foundation for Health Research (MSHFR) 150,000 CAD, Research Manitoba (RMB) 100,000 CAD, Research Nova Scotia (RNS) 100,000 CAD, Saskatchewan Health Research Foundation (SHRF) 50,000 CAD, and New Brunswick Research Foundation (NBHRF) Alberta Innovates (AI), Michael Smith Foundation for Health Research (MSFHR), Research Manitoba (RM), Research Nova Scotia (RNS), Saskatchewan Health Research Foundation (SHRF), and the New Brunswick Health Research Foundation (NBHRF) (https://www.canada.ca/en/institutes-health-research/news/2020/06/government-of-canada-and-provincial-partners-invest-more-than-109m-in-covid-19-research-details-of-the-funded-projects.html).

Research findings and data produced as a result of the funding will be shared rapidly and openly (in line with the joint statement on sharing research data and findings relevant to the novel coronavirus outbreak) to inform the global public health response and to help save lives.

Based on the last update from September 1st,2020, CIHR currently funds 332 COVID-19-related projects in two major areas: 1) Medical countermeasures focusing on: diagnostics, vaccines, therapeutics, clinical management, transmission dynamics and animal host modeling; and 2) Social and policy countermeasure projects focused on: studying the public health response and its impact, social dynamic communication and trust, coordination governance, and logistic. There are 6 (1.8% of all CIHR Covid-19 funded studies) operating grants with total of 2.47 M (1.4% of CIHR COVID-19 related funds) funding for projects addressing COVID-19 during reproduction (Table 2). Three are funded under the mental health and psychosocial/health behavioural research program (“Assessing and addressing the psychosocial impact of COVID-19 among pregnant woman and health care providers in Anhui, China”, 317,176 CAD; “Acceptability and Impact of Prenatal Internet Intervention for Maternal Mental Health in the COVID-19 Context”, 177,960CAD; and “Online 1-Day Cognitive Behavioural Therapy-Based Workshops for Postpartum Depression”, 199,567CAD. One reproduction-related project is funded under ‘nutrition in health research’ (“Can COVID-19 and maternal antibodies to SARS-CoV-2 be transmitted through human milk? Implications for breastfeeding and human milk banking”, 154,245CAD). In the field of Reproduction/pregnancy, CIHR currently funds two projects: “The COVID-19 Ontario Pregnancy Event (COPE) Network: Assessing the impact of pregnancy on maternal, fetal and newborn health”, 795,559CAD; and “Canadian Surveillance of COVID-19 in Pregnancy: Epidemiology, Maternal and Infant Outcomes”, 825,367CAD.

**Table 2:** Summary of CIHR funded studies addressing Covid-19 during reproduction

Research Area	Study Title	Investigator/Institution	Funding amount (CAD)	Operating Grant Program
Mental health and psychosocial/health behavioural research	Assessing and addressing the psychosocial impact of COVID-19 among pregnant woman and health care providers in Anhui, China	Shelby Yamamoto/ University of Alberta	$317,196	COVID-19 - Public health response and its impact
	Acceptability and Impact of Prenatal Internet Intervention for Maternal Mental Health in the COVID-19 Context	Deborah M Da Costa/ Research Institute of the McGill University Health Centre	$177,960	COVID-19 MH/SU - Developing Innovative Adaptations of Services/Delivery
	Online 1-Day Cognitive Behavioural Therapy-Based Workshops for Postpartum Depression	Ryan Van Lieshout/ McMaster University	$199,567	COVID-19 MH/SU - Developing Innovative Adaptations of Services/Delivery
Nutrition in health research	Can COVID-19 and maternal antibodies to SARS-CoV-2 be transmitted through human milk? Implications for breastfeeding and human milk banking	Deborah O’Connor/ Sinai Health System (Toronto) / University of Toronto	$154,245	COVID-19 Rapid Research FO - Clinical Mgmt/Health System Interventions
Reproduction/pregnancy	The COVID-19 Ontario Pregnancy Event (COPE) Network: Assessing the impact of pregnancy on maternal, fetal and newborn health	Darine El-Chaar; Marc-Andre Langlois/ Ottawa Hospital Research Institute	$795,559	COVID-19 Rapid Research FO - Clinical Mgmt/Health System Interventions
	Canadian Surveillance of COVID-19 in Pregnancy: Epidemiology, Maternal and Infant Outcomes	Deborah Money/ The University of British Columbia	$825,367	COVID-19 Rapid Research FO - Clinical Mgmt/Health System Interventions
Total	*N* = 6 (6/332, 1.8%)		$2,469,874	

*Mgmt*=Management

The COVID-19 Ontario Pregnancy Event (COPE) Network as a multisite study that will be assessing the impact of COVID-19 during pregnancy on maternal, fetal and newborn health. This study aims to assess the mother to infant and potential, vertical transmission of SARS-CoV-2 infection in pregnant women by analysis of prospectively collected maternal and neonatal biological samples (vaginal mucosa, amniotic fluid, placenta, cord blood, breast milk and neonatal NP swabs). They will also examine the serology and viral load in neonates and placenta to quantify the impact of SARS-CoV-2 on neonates.

Canadian COVID-19 In Pregnancy Surveillance (CANCOVID-Preg) Study (CANCOVID-Preg) will track maternal and infant outcomes among pregnant women with COVID-19 [138] by teams working in almost all provinces and territories across Canada, and will aid to the understanding of the epidemiology of COVID-19 in pregnancy, and will provide critical data to inform recommendations for pregnant women and their infants. The goals of the project are to determine the burden of COVID-19 among pregnant women in Canada, as well as maternal and neonatal outcomes associated with infection, including the possibility for vertical transmission. The data captured regionally is to be combined for a national dataset of cases of COVID-19 in pregnancy. Researchers will also contribute data to international collaborations, allowing for a more comprehensive global understanding of COVID-19 in pregnancy [138].

In addition, united efforts from a Group of 15 Canadian Research Universities, Compute Ontario, the University of Toronto and Canada’s science advisors, established a new CanCOVID (cancovid.ca), Canada-wide network of health, science and policy researchers to facilitate COVID-19 research collaboration. The aim is to provide rapid response network to expedite transdisciplinary communication and collaboration.

A Canadian initiative to study COVID-19 impact on ART is still to be conceived. Canada has invested in infrastructure to facilitate collaboration and research in reproduction, especially in ART, as evident from the numbers above, and calls for immediate action from basic science, clinical and psychosocial researchers from the field. In the U.S.A., the ASPIRE (Assessing the Safety of Pregnancy In the Coronavirus Pandemic) Study is a nationwide prospective cohort study of pregnant women and their offspring during the COVID-19 pandemic, that will include patients in the care of a reproductive medicine specialist who conceive spontaneously vs with ART, between March 1st and December 31st, 2020.

## Conclusions

COVID-19 pandemic has imposed unprecedented changes in all aspects of life and healthcare. Canada is currently at the beginning of the predicted second wave of COVID-19 infections. Active prevention with effective vaccine will likely be possible in early 2021, challenging the global response and management to be tailored and adjusted continuously and actively, as data becomes available.

Unlike previous pandemics, pregnant women do not appear to be at an increased risk for COVID-19 and are less likely to manifest COVID-19 related symptoms, compared to non-pregnant reproductive age women. Maternal outcomes are favourable, with severity of symptoms correlated to advanced maternal age and pre-existing comorbidities such as chronic cardio-pulmonary diseases, obesity and diabetes. Pregnant women have higher odds of needing ICU treatment for COVID-19, compared to non-pregnant women. Preterm birth rates (OR = 3.01, 95%CI 1.16–7.85) are higher in pregnant women with COVID-19 than in pregnant women without the disease, however this finding warrants caution, as there is no established causative relationship, and there is a possibility that the same risk factor predisposes to both COVID-19 and preterm birth. The high CS rate of 67–94% in COVID-19 infected pregnant women does not relate to obstetrical indication, but rather to infection control and management of the delivery and postnatal care. With respect to the impact of infection on maternal and fetal health in the first and second trimester, only a few reports are available, but preliminary data do not indicate an increased risk for miscarriage or fetal loss later on. Limited data on vertical transmission shows that it may be possible, occurring at a rate of ~ 3%, however, this finding must be interpreted with caution, since this data is for maternal infections occurring in the third trimester, and there are limitations of the methods used to detect SARS-CoV-2, that were not addressed in those studies. Future studies based on prospectively collected data and samples, analyzed with standardized protocols, during pregnancy and the neonatal period, including data on sequelae and long-term impact on health and development, will greatly add to current knowledge. This will allow for true risk estimation and provide guidance on epidemiologic and medical management of COVID-19 in this population. Observed shifts in the demographics of COVID-19 infections from predominantly over 60y at the beginning of pandemic to younger populations in the second wave, will likely provide the much-needed information on the reproductive age population.

The influence of COVID-19 on the female and male reproductive system needs further investigation. Future studies should include assessment of ovarian function in female patients during the acute and recovery phase. Furthermore, since there is no long term follow up, significant effects of this virus on reproductive function, cannot be excluded as yet. Since inflammation plays a significant role in the pathogenesis of some cases of premature ovarian insufficiency, it is currently unknown whether COVID-19 could induce a chronic systemic pro-inflammatory effect, which may impair ovarian reserve. Evidence so far suggests potential effects on the male reproductive system from COVID-19 infection. However, data regarding the effect on semen parameters needs further investigation. Future studies should focus on long-term effects on gonadal function in recovering patients. Fertility evaluation and follow-up in the months and years following recovery from COVID-19 infection should be considered for all COVID-19 male patients. Sperm cryopreservation could be considered for male patients at higher risk for infertility.

Given that the COVID-19 risk for reproductive age women appears to be low, in addition to reassuring initial evidence of low risk for pregnant patients and their fetuses, we feel that ART procedures and fertility treatments should not be delayed due to the pandemic. Adherence to practice guidance and preventative measures during COVID-19 pandemics should be strict. There is an immediate need for more research on ART during COVID-19 to address open questions on the impact of this virus on reproduction, and to allow for more informed patient care and counselling. The emotional impact of COVID-19 on infertile patients cannot be underestimated. Identifying patients at risk of psychological distress should be promoted by providing psychosocial education and training for health care professionals in the reproductive field. Future psychosocial research should focus on the possible long-term impact of COVID-19 on infertile patients in the coming years.

“Reproductive care is essential and reproductive medicine professionals are in a unique position to promote health and wellbeing. United efforts and collaboration are needed to gather together data and resources to enhance understanding of COVID-19 as it pertains to reproduction, pregnancy, and potential impact on the fetus and neonate. The lessons learned from current and future research in this area will be useful as humanity deals with future pandemics.” [110].

## Data Availability

Not applicable.

## Abbreviations

CoV: Coronavirus
SARS-CoV-2: Severe acute respiratory syndrome coronavirus-2
WHO: World Health Organization
LTC: Long term care
ART: Assisted Reproductive Technologies
CoVs: Coronaviruses
MERS: Middle East Respiratory Syndrome
S: Spike
M: Membrane
E: Envelope
N: Nucleocapsid
NTD: N-terminal domain
CTD: C-terminal (tail) domain
ACE2: Angiotensin converting enzyme 2
AT2: Alveolar epithelial type 2 cells
TMPRSS2: Transmembrane, serin protease-2
RAS: Renin-angiotensin system
Ro: Effective reproduction number
T: Testosterone
LH: Luteinizing hormone
HPG: Hypothalamic-pituitary gonadal
EPS: Expressed prostatic secretion
SGA: Small for gestational age
LBW: Low birth weight
CS: Caesarean Section
ASRM: American Society of Reproductive Medicine
CFAS: Canadian Fertility and Andrology Society
ESHRE: European Society for Human Reproduction and Embryology
IFFS: International Federation for Fertility Societies
ASPIRE: Assessing the Safety of Pregnancy In the Coronavirus Pandemic
SART: Society for Assisted Reproductive Technologies
CTLS: Cathepsine L-like cysteine protease
GW: Gestational weeks
RT-PCR: Reverse transcription-polymerase chain reaction
NP: Nasopharyngeal
aRR: Adjusted risk ratio
ICU: Intensive care unit
PPE: Personal protective equipment
